# Correction: Fringe proteins modulate Notch-ligand *cis* and *trans* interactions to specify signaling states

**DOI:** 10.7554/eLife.04998

**Published:** 2014-10-08

**Authors:** Lauren LeBon, Tom V Lee, David Sprinzak, Hamed Jafar-Nejad, Michael B Elowitz

LeBon L, Lee TV, Sprinzak D, Jafar-Nejad H, Elowitz MB. 2014. Fringe proteins modulate Notch-ligand *cis* and *trans* interactions to specify signaling states. *eLife* **3**:e02950. doi: 10.7554/eLife.02950. Published 25 September 2014

Hamed Jafar-Nejad and David Sprinzak were incorrectly listed as third and fourth authors respectively. The correct author order is: Lauren LeBon, Tom V Lee, David Sprinzak, Hamed Jafar-Nejad and Michael B Elowitz.

The article has been corrected accordingly.

