# Supplementary material for: Fringe proteins modulate Notch-ligand cis and trans interactions to specify signaling states
Source: eLife. 2014 Sep 25;3:e02950. doi: 10.7554/eLife.02950 (PMC4174579; doi:10.7554/eLife.02950)
Supplement: Supplementary file 1. — (A) Cell lines used in this work. (B) Plasmids used in this work. DOI: http://dx.doi.org/10.7554/eLife.02950.018 [file elife02950s001.docx]

**Supplementary materials**

**Supplementary File 1A. Cell lines used in this work**

| **Cell line name** | **Selection** | **Notes** | **Figure(s)** |
| --- | --- | --- | --- |
| CHO-K1 + TetR | Blasticidin 10μg/mL | **Background staining control** | **3, 4** |
| CHO-K1 + TetR + pEF-hN(ICD)-Gal4^esn^ | Blasticidin 10μg/mL, Geneticin 600μg/mL | **Background staining control**  **Notch expression = ΔΔCT ~2.7 (to β-actin)** | **3, 4** |
| CHO-K1 + TetR + pEF-hN(ΔICD)-Gal4^esn^ + TO-Dll1-cerulean | Blasticidin 10μg/mL, Geneticin 600μg/mL, Hygromycin 500μg/mL | **Notch1+Dll1** | **2, 3, 4** |
| CHO-K1 + TetR + pEF-hN(ΔICD)-Gal4^esn^ + TO-Jag1-cerulean | Blasticidin 10μg/mL, Geneticin 600μg/mL, Hygromycin 500μg/mL | **Notch1+Jag1** | **2, 3, 4** |
| CHO-K1 + TetR + pEF-hN(ΔICD)-Gal4^esn^ + TO-Dll1-cerulean + piggyBac-CMV-Lfng | Blasticidin 10μg/mL, Geneticin 600μg/mL, Hygromycin 500μg/mL, Puromycin 3μg/mL | **Notch1+Dll1+Lfng**  **Lfng expression = ΔΔCT 1.89 (to β-actin)** | **4** |
| CHO-K1 + TetR + pEF-hN(ΔICD)-Gal4^esn^ + TO-Dll1-cerulean + piggyBac-CMV-Mfng | Blasticidin 10μg/mL, Geneticin 600μg/mL, Hygromycin 500μg/mL, Puromycin 3μg/mL | **Notch1+Dll1+Mfng**  **Mfng expression = ΔΔCT 1.96 (to β-actin)** | **4** |
| CHO-K1 + TetR + pEF-hN(ΔICD)-Gal4^esn^ + TO-Dll1-cerulean + piggyBac-CMV-Rfng | Blasticidin 10μg/mL, Geneticin 600μg/mL, Hygromycin 500μg/mL, Puromycin 3μg/mL | **Notch1+Dll1+Rfng**  **Rfng expression = ΔΔCT 3.865 (to β-4actin)** | **4** |
| CHO-K1 + TetR + pEF-hN(ΔICD)-Gal4^esn^ + TO-Jag1-cerulean + piggyBac-CMV-Lfng | Blasticidin 10μg/mL, Geneticin 600μg/mL, Hygromycin 500μg/mL, Puromycin 3μg/mL | **Notch1+Jag1+Lfng**  **Lfng expression = ΔΔCT 2.13 (to β-actin)** | **4** |
| CHO-K1 + TetR + pEF-hN(ΔICD)-Gal4^esn^ + TO-Jag1-cerulean + piggyBac-CMV-Mfng | Blasticidin 10μg/mL, Geneticin 600μg/mL, Hygromycin 500μg/mL, Puromycin 3μg/mL | **Notch1+Jag1+Mfng**  **Mfng expression = ΔΔCT 1.72 (to β-actin)** | **4** |
| CHO-K1 + TetR + pEF-hN(ΔICD)-Gal4^esn^ + TO-Jag1-cerulean + piggyBac-CMV-Rfng | Blasticidin 10μg/mL, Geneticin 600μg/mL, Hygromycin 500μg/mL, Puromycin 3μg/mL | **Notch1+Jag1+Rfng**  **Rfng expression = ΔΔCT .525 (to β-actin)** | **4** |
| CHO-K1 + CMV- hN(ΔICD)-Gal4^esn^ +TO-Jag1-mCherry+UAS-H2B-citrine | Blasticidin 10μg/mL, Geneticin 600μg/mL, Hygromycin 500μg/mL, Zeocin 400μg/mL | **Notch1+Jag1 reporter cell line** | **5** |
| CHO-K1 + CMV- hN(ΔICD)-Gal4^esn^ +TO-Jag1-mCherry+UAS-H2B-citrine + pExchange-Lfng | Blasticidin 10μg/mL, Geneticin 600μg/mL, Hygromycin 500μg/mL, Zeocin 400μg/mL, Puromycin 3μg/mL | **Notch1+Jag1+Lfng reporter cell line**  **Lfng expression = ΔΔCT 2 (to β-actin)** | **5** |
| CHO-K1 + CMV- hN(ΔICD)-Gal4^esn^ +TO-Dll1-mCherry+UAS-H2B-citrine | Blasticidin 10μg/mL, Geneticin 600μg/mL, Hygromycin 500μg/mL, Zeocin 400μg/mL | **Notch1+Dll1 reporter cell line** | **5** |
| CHO-K1 + CMV- hN(ΔICD)-Gal4^esn^ +TO-Dll1-mCherry+UAS-H2B-citrine + pExchange-Lfng | Blasticidin 10μg/mL, Geneticin 600μg/mL, Hygromycin 500μg/mL, Zeocin 400μg/mL, Puromycin 3μg/mL | **Notch1+Dll1+Lfng reporter cell line**  **Lfng expression = ΔΔCT 2 (to β-actin)** | **5** |
| CHO-K1 + TetR + pEF-hN(ICD)-Gal4^esn^ + UAS-H2B-3x-citrine | Blasticidin 10μg/mL, Geneticin 600μg/mL, Zeocin 400μg/mL | **Pure receiver cell line** | **3-S2, 4** |

**Supplementary File 1B. Plasmids used in this work**

| **Construct name** | **Promoter** | **Gene** | **Mammalian selection** | **Role** |
| --- | --- | --- | --- | --- |
| pcDNA3- hN(ΔICD)-Gal4^esn^ | pEF | hN(ΔICD)-Gal4^esn^ | Neomycin  /Geneticin | Notch1 receptor in test cell lines |
| pcDNA3- hN(ΔICD)-Gal4^esn^ | CMV | hN(ΔICD)-Gal4^esn^ | Neomycin  /Geneticin | Notch1 receptor in movie cell lines |
| pcDNA5-TO-Dll1-cerulean | CMV-TO | Dll1-cerulean, short spacer sequence between Dll1 C-terminal and cerulean fluorescent protein | Hygromycin | Inducible Dll1 in test cell lines |
| pcDNA5-TO-Jag1-cerulean | CMV-TO | Jag1-cerulean, short spacer sequence between Jag1 C-terminal and cerulean fluorescent protein | Hygromycin | Inducible Jag1 in test cell lines |
| piggyBAC-CMV-Lfng | CMV | Lfng driven by CMV promoter | Puromycin | Constitutive Lfng in test cell lines |
| piggyBAC-CMV-Mfng | CMV | Mfng driven by CMV promoter | Puromycin | Constitutive Mfng in test cell lines |
| piggyBAC-CMV-Rfng | CMV | Rfng driven by CMV promoter | Puromycin | Constitutive Rfng in test cell lines |
| pExchange-Lfng | CMV | Lfng driven by CMV promoter | Puromycin | Constitutive Lfng in movie cell lines |
| pcDNA6-UAS-H2B-citrine/ pcDNA6-UAS-H2B-3x-citrine | UAS | H2B-citrine or H2B-3x-citrine (three citrines in tandem) | Zeocin | Fluorescent reporter for signaling from hN(ΔICD)-Gal4^esn^ |
